# Blood Pressure and Heart Rate Variability to Detect Vascular Dysregulation in Glaucoma

**DOI:** 10.1155/2015/798958

**Published:** 2015-10-01

**Authors:** Eva Charlotte Koch, Johanna Staab, Matthias Fuest, Katharina Witt, Andreas Voss, Niklas Plange

**Affiliations:** ^1^Department of Ophthalmology, RWTH Aachen University, Pauwelsstraße 30, 52072 Aachen, Germany; ^2^Department of Medical Engineering and Biotechnology, Ernst-Abbe-Hochschule Jena, 07745 Jena, Germany

## Abstract

*Purpose*. To investigate blood pressure and heart rate variability in patients with primary open-angle glaucoma (POAG) to detect disturbed blood pressure regulation. *Methods*. Thirty-one patients with POAG (mean age 68 ± 10 years) and 48 control subjects (mean age 66 ± 10 years) were included in a prospective study. Continuous blood pressure and heart rate were simultaneously and noninvasively recorded over 30 min (Glaucoscreen, aviant GmbH, Jena, Germany). Data were analyzed calculating univariate linear (time domain and frequency domain), nonlinear (Symbolic Dynamics, SD) and bivariate (Joint Symbolic Dynamics, JSD) indices. *Results*. Using nonlinear methods, glaucoma patients were separated with more parameters compared to linear methods. In POAG, nonlinear univariate indices (pW113 and pW120_Sys) were increased while the indices pTH10_Sys and pTH11_Sys reflect a reduction of dominant patterns. Bivariate indices (JSDdia29, JSDdia50, and JSDdia52; coupling between heart rate and diastolic blood pressure) were increased in POAG. The optimum set consisting of six parameters (JSDdia29, JSDdia58, pTH9_Sys, pW231, pW110_Sys and pW120_Sys) revealed a sensitivity of 83.3% and specificity of 80.6%. *Conclusions*. Nonlinear uni- and bivariate indices of continuous recordings of blood pressure and heart rate are altered in glaucoma. Abnormal blood pressure variability suggests disturbed autonomic regulation in patients with glaucoma.

## 1. Introduction

Glaucoma is a multifactorial chronic progressive disease, characterized by the loss of ganglion cells which leads to typical damage of the optic nerve and to visual field loss. The pathogenic concepts of glaucoma may be divided into a mechanical, pressure-related, and a vascular approach. It is well established that the main risk factor for glaucoma is individually elevated intraocular pressure. In addition, systemic vascular factors like arterial hypertension and hypotension, cardiovascular diseases, vasospasms, and others have been identified to play a significant role in the disease as well as impaired ocular blood flow [1–3].

In this context, disturbed vascular regulation may increase the susceptibility of the optic nerve and the ganglion cells to fluctuations in ocular perfusion pressure. Systemic blood pressure regulation and local mechanisms (autoregulation) need to maintain ocular blood flow at a constant level despite changes in perfusion pressure [4, 5]. It has been shown that low ocular perfusion pressure is a risk factor for the prevalence of glaucoma. Abnormal perfusion and the following ischemia of the optic nerve are supposed to lead to glaucomatous damage [6, 7]. Disturbed autoregulation was observed in several studies in glaucoma [4, 8, 9]. The mechanisms may be related to primary vascular dysregulation, endothelial dysfunction, astrocyte activation, or increased intraocular pressure [7, 10]. In a previous study on 24 h blood pressure monitoring in glaucoma, an increased night-time blood pressure variability was found suggesting disturbed systemic blood pressure regulation [11].

The present prospective pilot study investigates the autonomic blood pressure regulation in patients with primary open-angle glaucoma (POAG) and controls. Systolic and diastolic blood pressure and heart rate variability was noninvasively and continuously assessed to characterize systemic vascular dysregulations using linear and nonlinear methods. The hypothesis is that POAG patients exhibit a different pattern of blood pressure and heart rate variability compared to controls as defined by using nonlinear analyzing methods.

## 2. Methods

Thirty-one patients with POAG and 48 age-matched controls were included in a prospective pilot study. All patients with POAG had a detailed ophthalmological examination; intraocular pressure was measured using Goldmann applanation tonometry. Patient's history was explored with special interest on cardiovascular risk factors (diagnosis of treated hypertension, arterial hypotension, history of cardiovascular events, nicotine abuse, and obesity). The systemic medications were recorded, but only the status of treated arterial hypertension was included in the analysis. Participants were recruited from the Department of Ophthalmology at the University of Aachen. Informed consent was obtained from all subjects. Adherence to the Declaration of Helsinki for research involving human subjects is confirmed. Ethics approval was granted by the committee of ethics at the University of Aachen.

To measure cardiovascular biosignals, blood pressure, and electrocardiogram, the diagnostic system “Glaucoscreen” (aviant GmbH, Jena, Germany) was used.

**Figure 1:**
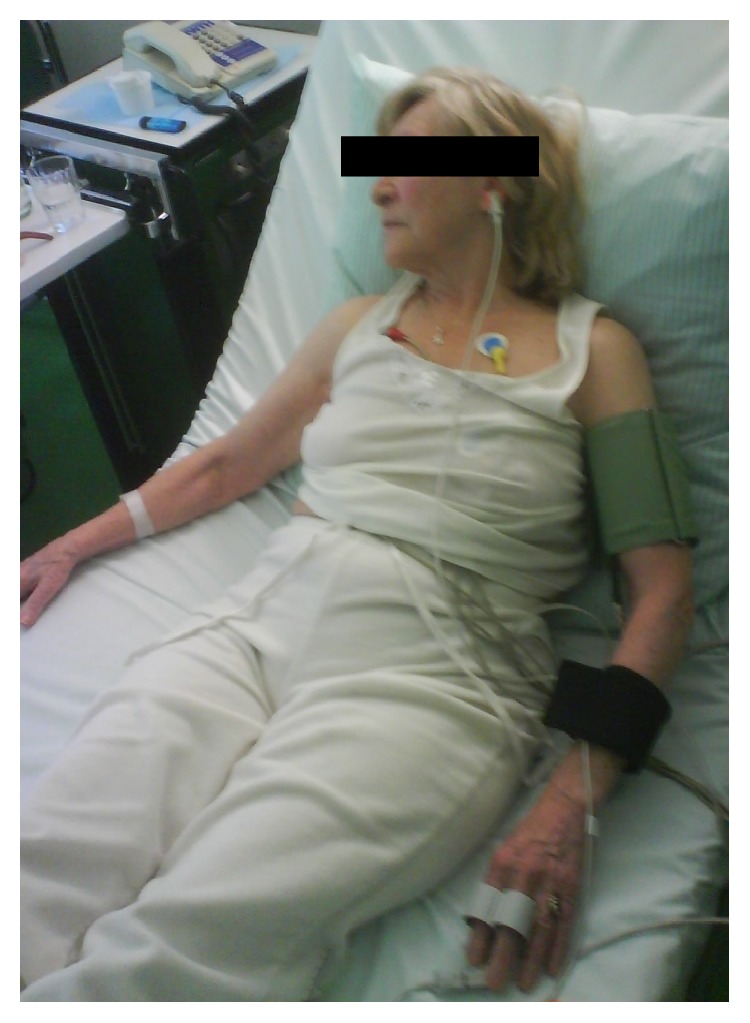
Simultaneous and continuous multichannel registration of diastolic and systolic blood pressure and heart rate in a lying position at rest.

**Figure 2:**
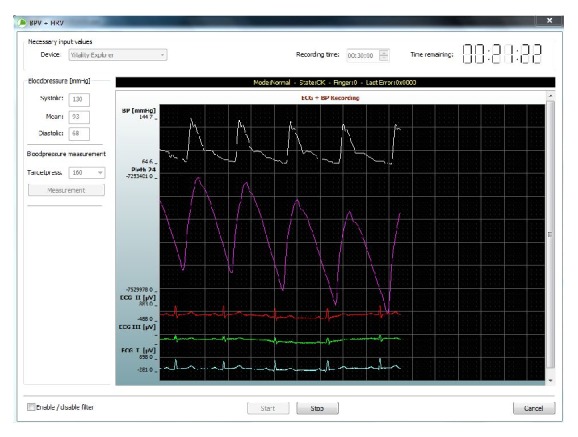
Synchronous ECG and blood pressure time series were continuously recorded and computer controlled.

Synchronous ECG and blood pressure time series were continuously recorded over a period of 30 minutes in a lying position at rest using Glaucoscreen (aviant GmbH, Jena, Germany); see Figures 1 and 2. The method focusses on the detection of abnormal fluctuations of the cardiovascular system and the mechanisms of systemic autonomic regulation in patients without any physical activity. This system allows simultaneous and continuous multichannel registration of diastolic and systolic blood pressure and heart rate. Participants avoided activities that could alter the blood pressure or heart rate 30 min before examination. Nicotine or caffeine intake was not allowed on the day of the examination. For preparing the recording, at first electrodes were fixed on subject's body to record the electrocardiogram. For calibration, blood pressure was measured once before starting the recording using the upper arm. During the period of 30 minutes, blood pressure was measured continuously at two fingers, applying the noninvasive CNAP OEM Module (CNSystems Medizintechnik AG, Austria). The recording was started automatically controlled by the computer software. All recordings were performed under resting conditions (supine position, quiet environment, and the same location) and patients were instructed to lie calmly and to avoid speaking.

Systemic blood pressure variability (BPV) and heart rate variability (HRV) were analyzed offline (Ernst-Abbe-Hochschule Jena, Department of Medical Engineering and Biotechnology, Jena, Germany). For data preprocessing, the time series of successive beat-to-beat intervals (BBIs) and of systolic as well as diastolic pressure values were extracted. HRV is the BBI length variability also called RR variability (where R represents the peak of the QRS complex of the ECG complex and RR is a time interval between two successive R peaks) whereas BPV represents the variability of successive systolic or diastolic blood pressure values corresponding to the related heart beats. In a further step, artefacts and/or ectopic beats and other disturbances (either R peaks recognized incorrectly or R peaks generated not in sinus rhythm) were detected within the RR time series and replaced (applying an adaptive filter) by interpolated “normal” beats to generate normal-to-normal (NN) beat time series representing normal sinus rhythm of the heart.

HRV and systolic and diastolic BPV standard parameters were calculated from time (Table 1) and frequency domain (Table 2) according to the Task Force of the European Society of Cardiology and the North American Society of Pacing and Voss et al. [12, 13].

In addition to these linear analysis methods, Symbolic Dynamics (SyD) and Joint Symbolic Dynamics (JSD), two nonlinear methods were applied in this study. Results of SyD analysis have been shown to be sufficient for the investigation of complex systems and describe dynamic aspects within time series. SyD is a nonlinear method which describes the global short- and long-term dynamics of beat-to-beat variability on the basis of symbolization and was introduced by Kurths and Voss et al. [14, 15]. The SyD method converts the NN interval time series into an alphabet of four predefined symbols (0, 1, 2, and 3) according to the transformation rules based on consecutive comparison of successive NN intervals.

The symbols “0” and “2” reflect slight deviations (<10% increase, resp., 10% decrease) from the mean NN interval, and the symbols “1” and “3” reflect stronger deviations (>10% increase, resp., 10% decrease) from the mean NN interval.

Then, the symbol strings are transformed into word series where each word consists of three successive symbols. This leads to a range of 64 different word types (xxx = 000, 001,…, 333). Then, estimates from the word distribution using the probability of occurrence (pWxxx) of each word type within NN interval time series are calculated (the summarized probability of all word types is normalized to 1).

On the basis of these word types, the number of all word types with a probability of occurrence of more than yyy percent (pTHyyy for HRV and _ pTHyyy _Sys/Dia for BPV) was separately counted (e.g., pTH13_Sys means the number of word types with a probability of occurrence greater than 13% in systolic blood pressure time series).

The SyD indices were all calculated for the heart rate and systolic and diastolic blood pressure time series.

**Figure 3:**
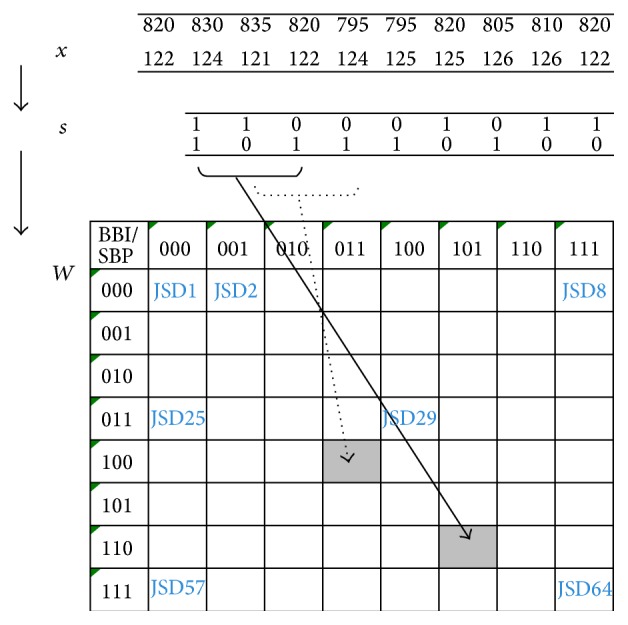
The method of Joint Symbolic Dynamics (JSD) quantifies the short-term bivariate nonlinear behavior (coupling) of blood pressure and heart rate. JSD transforms BBIs (beat-to-beat intervals) and diastolic or systolic blood pressure time series into symbol sequences of different words (3 successive symbols are one word) according to the pattern of change (increase or decrease) [14]. Top: *x* as time series of BBI (in ms) and systolic blood pressure values (in mmHg). Middle: *s* as symbol vector. Bottom: *W* as word type matrix with BBI (columns); SP, systolic blood pressure (rows). With JSD1…JSD64 as coupling indices.

The JSD (Figure 3) [16] is a bivariate method investigating interactions between BBI time series and systolic or diastolic time series. JSD was applied to quantify the short-term bivariate nonlinear behavior of the cardiovascular system. Similar to the SyD, JSD transforms BBIs, diastolic, and systolic blood pressure time series into symbol sequences of different words *W* according to the transformation rules using an alphabet *A* = {0,1}. Thereby, symbol “1” represents increasing values (the actual value is greater than the previous one) and symbol “0” decreasing and unchanged values (the actual value is less than or equal to the previous one) applying a threshold level equal to zero.

Afterwards, short patterns (words of length consisting of 3 symbols) were formed (*k* = 64) in detail; see Figure 3.

The analysis method of JSD included the evaluation of 64 parameters for characterization of systolic blood pressure interaction with heart rate and 64 parameters for the interaction of diastolic blood pressure and heart rate.

### 2.1. Statistical Analysis

The analysis was performed on the basis of the HRV/BPV indices (time and frequency domain, methods from nonlinear dynamics). The nonparametric Mann-Whitney* U* test (SPSS 21) was applied for statistical analysis to figure out significant (*p* < 0.05) and highly significant (*p* < 0.001) parameters differentiating between patients with glaucoma and controls. For highly significant parameters, the multivariate stepwise discriminant function analysis was performed to calculate the specific sensitivity and specificity and the area under the receiver operating characteristics (ROC) curve (AUC) applying the best set of six parameters.

## 3. Patients

Thirty-one patients with POAG and 48 age-matched volunteers were included in this prospective pilot study. Patients with POAG had a glaucomatous excavation of the optic disc and glaucomatous visual field defects as defined by the European Glaucoma Society [17]. The diagnostic criteria for glaucomatous visual field loss are as follows. Field loss was considered significant when (a) glaucoma hemifield test was abnormal, (b) 3 points were confirmed with *p* < 0.05 probability of being normal (one of which should have *p* < 0.01), not contiguous with the blind spot, or (c) corrected pattern standard deviation (CPSD) was abnormal with *p* < 0.05. All parameters were confirmed on two consecutive visual fields performed with Humphrey Field Analyzer. All patients with POAG had IOP values above 21 mmHg without treatment in their medical history. Visual field examinations were performed with the Humphrey Field Analyzer (Model 750, Humphrey-Zeiss, San Leandro, California, SITA program 24-2).

The control subjects did not have any ophthalmologic disease, showed IOP values below 22 mmHg, and did not receive any topical treatment. Visual field examinations did not reveal any significant visual field loss. Visual field parameters (mean deviation (MD) and pattern SD (PSD), Humphrey Visual Field Analyzer) were within normal range and the glaucoma hemifield test was within normal limits. Healthy controls presented in funduscopy a normal optic nerve head appearance (no thinning or notching of neuroretinal rim, no bared circumlinear vessels, and no disc hemorrhages).

Volunteers and patients with POAG with an acute cardiovascular or cerebrovascular event within the last 6 months or with known heart rhythm disorders were excluded from this study.

Patients with glaucoma and controls were matched for age, sex, and treated arterial hypertension. All patients with glaucoma were on topical IOP lowering therapy that might influence the results.

## 4. Results

Thirty-one patients with POAG (mean age 66 ± 10 years; 17 men, 14 women) and 48 control subjects (mean age 68 ± 10 years; 24 men, 24 women) were included in this study.

Patients with POAG had on the right eye a mean IOD of 15 ± 3 mmHg (minimum 8 mmHg, maximum 36 mmHg) and on the left eye a mean IOD of 17 ± 5 mmHg (minimum 10 mmHg, maximum 33 mmHg). IOD of the healthy controls was never above 21 mmHg; mean IOD for the right eye was 15 ± 3 mmHg and for the left eye 14 ± 3 mmHg. All patients suffering from POAG were on topical IOP lowering medications. Seventeen patients used carbonic anhydrase inhibitors, 24 patients ß-blockers, 6 patients brimonidine, and 19 patients prostaglandins. Twenty-nine patients with POAG and 23 controls confirmed in their medical history treated arterial hypertension. The clinical data (systemic vascular risk factors by medical history) of both groups are shown in Table 3.

In the group of POAG, patients had a mean diastolic blood pressure of 89 ± 9 mmHg and a mean systolic blood pressure of 145 ± 17 mmHg. The controls showed a mean diastolic blood pressure of 86 ± 12 mmHg and a mean systolic blood pressure of 147 ± 17 mmHg. Mean heart rate in the group of POAG was 68 ± 10/min; healthy controls showed a mean heart rate of 67 ± 10/min. The mean as well as standard deviation of the heart rate and diastolic and systolic blood pressure did not significantly differ between the two investigated groups.

Looking at the time series of heart rate and systolic blood pressure, more parameters belonging to the nonlinear analysis methods (SyD) were able to significantly separate control subjects and patients with POAG compared to the time series of diastolic blood pressure, where only one SyD parameter could separate the two groups significantly (Table 4).

Applying the method of JSD, more significant parameters were detected when analyzing the interaction of the time series diastolic blood pressure and heart rate compared to the interaction of time series systolic blood pressure and heart rate. The univariate indices pW113 and pW120_Sys (probability of occurrence of the specific word types: 113 for beat-to-beat intervals and 120 for systolic blood pressure) were increased in POAG. That means an increase of patterns with a valley-like behavior of heart rate patterns and an increase of systolic BPV patterns with a start of a plateau phase.

The univariate indices pTH10_Sys and pTH11_Sys (number of systolic BPV word types with a probability of occurrence higher than 10, resp., 11 percent) reflect a reduction of dominant patterns at the expense of an increased probability of occurrence of other word types (an increase of pTH3). That means that POAG exhibit a higher systolic BPV than controls.

Finally, the indices JSDdia29, JSDdia50, and JSDdia52 were increased in POAG. These indices characterize the coupling between heart rate and diastolic blood pressure. Interestingly all these indices may be found directly or in neighborhood to the word types representing diametric behavior (e.g., 011,100 or 110,001). These word types demonstrate a behavior oppositional to the typical baroreflex response (e.g., 001,001 or 110,110).

Overall, only single linear indices from time and frequency domain analysis showed significant differences between groups. All significant parameters are shown in Tables 4 and 5.

The optimum set consisting of six parameters (JSDdia29, JSDdia58, pTH9_Sys, pW231, pW110_Sys, and pW120_Sys) revealed a sensitivity of 83.3%, a specificity of 80.6%, and an AUC of 82.3%.

## 5. Discussion

Ocular blood flow is an important factor in glaucomatous optic neuropathy and, together with ocular perfusion pressure, is directly affected by systemic blood pressure [3, 18]. The influence of systemic blood pressure on glaucomatous optic neuropathy has been investigated in several studies before [1, 11, 19, 20]. Systemic blood pressure has different fluctuation rhythms under physiological conditions. Systemic blood pressure has different fluctuation rhythms under physiological conditions, that is, seasonal variability with lower blood pressure values in winter times, short-time fluctuation patterns during day and night and the physiological night-time blood pressure depression [11]. There is evidence that excessive dipping could be associated with development or progression of glaucoma [21]. Sung et al. examined the relationship between 24 h mean ocular perfusion pressure and visual field progression in patients with normal tension glaucoma. In that retrospective study, 101 patients had at least a 4-year follow-up, and blood pressure and IOP were evaluated over 24 hours in each patient. Sung et al. showed that elevated 24 h mean arterial pressure and increased 24 h mean ocular perfusion pressure fluctuations were a significant risk factor for glaucoma progression [19].

Local blood flow of the optic nerve head is organized by autoregulation [22]. Autoregulation is the physiological phenomenon in which the resistance changes dynamically to keep blood flow at a constant level which is required by the local and metabolic activity despite changes in perfusion pressure. In healthy subjects, retinal blood flow is autoregulated to provide a constant blood flow regardless of changes in ocular perfusion pressure [4, 5]. Earlier studies have suggested that glaucoma patients show abnormal autoregulation especially in response to acute changes in ocular perfusion pressure [21, 22]. In glaucoma, autoregulatory dysfunction may be related to fluctuations in ocular perfusion pressure, via changes in either systemic blood pressure or intraocular pressure, leading to changes in retinal and optic nerve head perfusion [21, 23, 24]. The concept of altered ocular blood flow has been postulated to be a major component of glaucoma pathogenesis in normal tension glaucoma. However, disturbed blood flow has been found to be relevant in primary open-angle glaucoma also [3, 25–27].

In addition to the concept of autoregulation, systemic blood pressure and heart rate are influenced by systemic mechanisms of regulation. The concept of systemic vascular dysregulation in glaucoma affecting recurrent ischemic episodes of the optic nerve caused by an impaired capacity to compensate low perfusion pressures has been described before [10, 21]. The concept of disturbed blood flow regulation in glaucoma has been investigated in various studies using different approaches provoking the capability of systemic and local mechanisms of blood flow regulation. Stimuli such as carbon dioxide, oxygen, cold stress, isometric exercise, brachial arterial occlusion, or light flicker were used to examine blood flow regulation in patients with normal tension and primary open-angle glaucoma [9, 22, 28]. However, until today, no standard method to measure vascular dysregulation has been established. In contrast to provocation methods that focus on autoregulation of ocular blood flow, the concept of the present study is to quantify the extent of defective or abnormal systemic vascular regulation of blood pressure and heart rate without any stimulus given. Using this approach, the autonomic system may be characterized without any influence ab externo.

In our study, blood pressure and heart rate variability was noninvasively assessed to characterize systemic vascular dysregulation in glaucoma patients and controls. Nonlinear analyzing parameters of blood pressure and heart rate data were significantly different. The nonlinear model was designed to account for complex interactions of the continuously gained values characterizing systemic blood pressure and heart rate variability and dysregulation. In contrast, only singular linear indices from time and frequency domain showed significances. However, these indices could not contribute to the discrimination between glaucoma and controls in the same level as the nonlinear methods SyD and JSD. The optimum set consisting of 6 indices revealed a sensitivity of 83.3% and a specificity of 80.6%. Interestingly, in this set only, indices from nonlinear dynamics (Symbolic Dynamics) were included. In this context, we should emphasize that we do not believe that the identification of abnormal blood pressure or heart rate patterns would probably be a tool to identify glaucoma patients. But the measurement of systemic autonomic dysregulation might be an important method to identify patients with an increased risk for progression due to an impaired capacity of optic nerve head perfusion.

Methods of heart rate variability (HRV) and BPV based on nonlinear system theory and beat-to-beat dynamics have gained recent interest as they may reveal dedicated changes of autonomic regulations. These methods have been already used in other studies, investigating risk estimation for sudden cardiac death in patients with cardiomyopathy [15] or examining heart rate variability in normal pregnancy [16].

There are various types of different fractal scaling measures, complexity measures, power law analysis, measures of Symbolic Dynamics, turbulence, and acceleration/deceleration of heart rate and blood pressure and they have been studied in various patient populations [29, 30].

In this study, especially indices from SyD (univariate) and JSD (bivariate, coupling) exhibit significant impairments in the cardiovascular regulation in glaucoma patients. These results support the idea that glaucoma is not just a process involving the eye but may be a manifestation of a more generalized autonomic dysfunction that is in agreement with the findings of Brown et al. and others [31, 32]. Andrikopoulos et al. [33] summarized that PEX syndrome may be linked to impaired heart and blood vessels function, systemic and ocular blood flow changes, altered parasympathetic vascular control and baroreflex sensitivity, increased vascular resistance and decreased blood flow velocity, arterial endothelial dysfunction, high levels of plasma homocysteine, and arterial hypertension. These partly complex linked impairments might be a reason for the found coupling impairments in POAG in this study. In general, an increased blood pressure variability is associated with cardiovascular disorders [34]. An increased variability of systolic blood pressure represents also a strong predictor of early carotid atherosclerosis progression in general population [35]. In a 3-year follow-up study, progression of intima-media wall thickness was significantly greater in the patients with increased systolic BPV even after adjustment for other risk factors. Moreover, especially an increased daytime systemic BPV was associated with a greater risk of cardiovascular events [35]. We could confirm such an increased systemic BPV in this study. The reduced values of pTH10 and pTH11 (a lower number of dominating word types leading to a more broadly distributed variability) and the increased values of pWsys120 (representing an increased number of alternating patterns of the systolic blood pressure) are typical signs of an increased BPV. These patterns are related to an increased number of downregulations of the heart rate and to more temporal limitations of blood pressure increases. Both together might reflect an increased number of baroreflex activities to short-term down- regulated systolic blood pressure.

Even the sensitivity of baroreflex control of heart rate is depressed in glaucoma patients [31]; the number of tachycardic baroreflexes seems to be increased. Higher values of JSDdia29, JSDdia50, and JSDdia58 indicate enhanced occurrences of baroreflex regulations (couplings between diastolic blood pressure and heart rate) and, therefore, impaired short-term blood pressure regulation in glaucoma patients. Diastolic blood pressure variability and baroreflex characterize the short-term behavior of the cardiovascular system and are mainly determined by respiratory influences on the blood pressure and heart rate. Therefore, coupling analyses might uncover impairments of the autonomic blood pressure regulation [36].

However, it might be too early for this method for a more specific interpretation of the data. Further studies have to show if our pilot results can be reproduced in a larger scaled investigation. A major limiting factor of this prospective study is the possible influence of other systemic vascular diseases and topical and systemic medications possibly affecting ocular blood flow. A large controlled prospective study would be appropriate to investigate such confounding factors and to validate the results of this study. Secondly, this approach has to be investigated in patients with normal tension glaucoma as well, to learn if the same or other parameters will be found to be altered in these patients.

## 6. Conclusions

In conclusion, these alterations in blood pressure variability and coupling with heart rate suggest modified autonomic regulation due to a vascular dysfunction in patients suffering from glaucoma. The importance of the vascular influence for the pathogenesis of glaucoma is again emphasized by this study. Further studies need to show if the method is valuable to identify systemic autonomic dysfunction in glaucoma. Patients with systemic autonomic dysfunction might be at higher risk for progression of the disease due to a higher susceptibility of the optic nerve to fluctuations in intraocular pressure or ocular perfusion pressure.

## Figures and Tables

**Table 1:** Description of parameters calculated by time domain analysis. Parameters were calculated for heart rate and systolic and diastolic blood pressure.

Method	Parameter	Description
Time domain	MEANNN	Mean value of all NN intervals
	SDNN	Standard deviation of all NN intervals
	CVNN	Coefficient of variation of all NN intervals
	SDANN1	Standard deviation of the averages of NN intervals in all 1-minute segments of the entire recording
	SDANN5	Standard deviation of the averages of NN intervals in all 5-minute segments of the entire recording
	SDANN10	Standard deviation of the averages of NN intervals in all 10-minute segments of the entire recording
	RMSSD	Square root of the mean squared differences of successive NN intervals
	PNN50	Proportion derived by dividing the number of interval differences of successive NN intervals greater than 50 ms by the total number of NN intervals
	PNN100	Proportion derived by dividing the number of interval differences of successive NN intervals greater than 100 ms by the total number of NN intervals
	PNN200	Proportion derived by dividing the number of interval differences of successive NN intervals greater than 200 ms by the total number of NN intervals
	PNNL10	Portion of NN interval differences <10 ms in all NN intervals
	PNNL20	Portion of NN interval differences <20 ms in all NN intervals
	PNNL30	Portion of NN interval differences <30 ms in all NN intervals
	PNNL50	Portion of NN interval differences <50 ms in all NN intervals
	RENYI2	Renyi entropy of the histogram with (order) alpha = 2
	RENYI4	Renyi entropy of the histogram with alpha = 4
	RENYI025	Renyi entropy of the histogram with alpha = 0.25
	SHANNON	Shannon entropy of the histogram

**Table 2:** Description of parameters calculated by frequency domain analysis. Parameters were calculated for heart rate and systolic and diastolic blood pressure.

Method	Parameter	Description
Frequency domain	ULF	Power (=variability) in “ultra low frequency” range (0–0.0033 Hz)
	VLF	Power (=variability) in “very low frequency” range (0.0033–0.04 Hz)
	LF	Power (=variability) in “low frequency” range (0.04–0.15 Hz)
	HF	Power (=variability) in “high frequency” range (0.15–0.4 Hz)
	XHF	Extended high frequency band from 0.15 to 0.6 Hz
	XF	Frequency band from 0.12 to 0.18 Hz
	P	Total power density spectra (variance of all NN intervals ≤0.4 Hz)
	LF/HF	Ratio of LF and HF
	LF/P	Ratio of LF and P
	HF/P	Ratio of HF and P
	XHF/PX	Ratio of XHF and the extended total power (variance of all NN intervals ≤0.6 Hz)
	VLF/P	Ratio of VLF and P
	ULF/P	Ratio of ULF and P
	(ULF + VLF + LF)/P	Ratio of (ULF + VLF + LF) and P
	(ULF + VLF)/P	Ratio of (ULF + VLF) and P
	UVLF	Sum of ULF, VLF, and LF (≤0.15 Hz)
	LFN	Normalized low frequency
	HFN	Normalized high frequency

**Table 3:** Clinical data of patients with POAG and control subjects.

	POAG	Controls
Age (years)	68 ± 10	66 ± 10
Treated arterial hypertension	18/31	17/48
Diabetes mellitus	4/31	3/48
Cardiovascular or Cerebrovascular events	5/31	0/48
Adiposity (body mass index >30 kg/m^2^)	1/31	9/48
Hypotonia/Raynaud's Phenomenon	5/31	0/48
Nicotine abuse	4/31	11/48

**Table 4:** Analysis methods and amount of significant parameter (*p* < 0.05) for controls versus POAG for each time series.

Time series and used analysis method	Amount of significant parameter controls versus POAG
Heart rate variability	
Time domain	0 out of 18
Frequency domain	1 out of 18
Symbolic Dynamics	5 out of 99

Systolic blood pressure	
Time domain	1 out of 18
Frequency domain	0 out of 18
Symbolic Dynamics	11 out of 99

Diastolic blood pressure	
Time domain	0 out of 18
Frequency domain	0 out of 18
Symbolic Dynamics	1 out of 99

Systolic JSD	1 out of 64

Diastolic JSD	7 out of 64

**Table 5:** Significant parameters (*p* value, mean value, and standard deviation) separating controls versus patients with POAG according to time series and analysis methods.

Time series and analysis method	Significant parameters	Controls versus POAG	Controls		POAG	
			Mean	Std.	Mean	Std.
Heart rate						
Frequency domain	LFP	**4.81E − 02**	0.2025	0.0986	0.2534	0.1222

Heart rate						
Symbolic Dynamics	pW031	4.71*E* − 02	0.0001	0.0002	0.0001	0.0003
	pW113	**3.93E − 03**	0.0000	0.0001	0.0004	0.0016
	pW231	1.01*E* − 02	0.0001	0.0003	0.0003	0.0004
	pW310	3.45*E* − 02	0.0001	0.0002	0.0003	0.0006

Blood pressure systolic						
Time domain	renyi4_Sys	4.26*E* − 02	1.9573	0.4239	1.7789	0.4075

Blood pressure systolic						
Symbolic Dynamics	pW003_Sys	3.98*E* − 02	0.0006	0.0013	0.0012	0.0021
	pW011_Sys	2.00*E* − 02	0.0184	0.0082	0.0150	0.0073
	pW110_Sys	2.42*E* − 02	0.0181	0.0079	0.0150	0.0076
	pW120_Sys	**5.07E − 04**	0.0005	0.0010	0.0011	0.0015
	pTH3_Sys	4.20*E* − 02	7.3333	1.6417	8.0323	1.6829
	pTH8_Sys	1.13*E* − 02	3.1458	1.0717	2.5484	0.7229
	pTH9_Sys	1.59*E* − 02	2.8125	1.0033	2.2903	0.5287
	pTH10_Sys	**1.50E − 03**	2.5000	0.9453	2.0323	0.1796
	pTH11_Sys	**5.00E − 03**	2.3333	0.9070	1.8710	0.4995
	pTH12_Sys	1.51*E* − 02	2.1875	0.6410	1.8710	0.4995
	pTH13_Sys	4.71*E* − 02	2.1042	0.6601	1.8387	0.5829

Blood pressure diastolic						
Symbolic Dynamics	pW202_Dia	1.55*E* − 02	0.0212	0.0121	0.0280	0.0146

Joint Symbolic Dynamics systolic blood pressure, heart rate	JSD30	3.23*E* − 02	0.0116	0.0067	0.0141	0.0059

Joint Symbolic Dynamics diastolic blood pressure, heart rate	JSDdia29	**9.74E − 03**	0.0283	0.0145	0.0417	0.0239
	JSDdia33	2.17*E* − 02	0.0183	0.0097	0.0130	0.0069
	JSDdia37	3.58*E* − 02	0.0107	0.0112	0.0072	0.0044
	JSDdia50	**8.11E − 03**	0.0276	0.0145	0.0392	0.0217
	JSDdia52	**9.23E − 03**	0.0118	0.0085	0.0169	0.0111
	JSDdia53	2.49*E* − 02	0.0197	0.0136	0.0137	0.0087
	JSDdia58	2.62*E* − 02	0.0055	0.0040	0.0083	0.0055
